# The Diagnosis of Urethral Stricture of Large Calibre

**Published:** 1889-09

**Authors:** R. W. Stewart

**Affiliations:** Physician to Mercy Hospital, Pittsburg, Pa.


### THE DIAGNOSIS OF URETHRAL STRICTURE OF LARGE CALIBRE.*

R. W. STEWART, M. D., M. R. C. S., PHYSICIAN TO MERCY HOSPITAL, PITTSBURG, PA.

A prominent authority on genito-urinary diseases says: "The least contraction at any point in the urethral canal has been demonstrated as capable of causing the indefinite continuance of an urethral discharge and even of establishing it *de novo* without venereal contact." The same authority also says: "Chronic urethral discharges, commonly called gleet, are the signals which nature hangs out to notify the intelligent surgeon that an obstruction to the normal working of the muscular apparatus of the urethra has occurred, that plastic material laid down in the antecedent inflammatory condition has begun to contract the normal urethral calibre, whether it be twenty or forty millimetres in circumference and that nothing short of a complete restoration of the normal calibre of the canal will afford a permanent cure." (Otis, "Stricture of the male urethra," pages 20 and 75.) Those who are accustomed to the treatment of urethral diseases are aware that the treatment of gleet constitutes perhaps the most important as well as the most troublesome part of urethral surgery; and if, as there are good reasons for believing, the successful treatment of gleet consists, in the majority of cases, of the removal from the urethra of some contraction in its calibre, which keeps the adjacent mucous membrane in a condition of chronic inflammation, it will be evident how important it is that some means should be placed at our disposal by which urethral stricture may be readily detected and accurately located.

For this purpose various instruments have been devised; those in general use are the blunt pointed steel sound, the bulbous bougie and the urethrometer.

The blunt pointed steel sound, as recommended by Sir Henry

*Read at Allegheny Co. Med. Society, June 18, 1889.

Thompson, is doubtless less useful for the detection of stricture of small calibre, through which only small instruments will pass. But these strictures are not those with which we have most to contend with; it is the stricture of large calibre, through which a medium sized steel sound will pass without perhaps a noticeable obstruction. The blunt pointed steel sound is certainly inadequate as a means of detecting strictures of large calibre, and in the presence of superior instruments should be relegated to obscurity.

The bulbous bougies are, in many respects, superior to the steel sound, but to their use may be urged several serious objections, which they have in common with the steel sounds. It is necessary to have a complete set of bougies, as each bougie only gauges a particular size. Where there is more than one contraction of the urethra, should the posterior contraction be less than the anterior, the bulbous bougie will not indicate its presence until the anterior contraction is dilated sufficiently to pass an instrument the size of the posterior stricture, and a contraction of the meatus, so commonly found, renders them useless, until the meatus is cut. Another objection to these instruments is the necessity of trying bougie after bougie until the proper size is obtained.

To obviate these objections the urethrometer was devised. There are several varieties of this instrument, all agreeing, however, in their general construction and method of using. This instrument has a bulbous extremity, which can be expanded to any desired extent by means of a screw at the handle. An index at the handle indicates in millimetres the size of the expanded bulb. The urethrometer is free from the objections of the previous instruments, in the fact that it can be adjusted so as to measure any stricture of large calibre, and that a contracted meatus or a narrow anterior stricture forms no obstacle to the detection of deeper strictures. It must be admitted that the urethrometer is an improvement on the sound and the bougie, but it is perhaps better in theory than in practice, for in practice it has serious objections. Following the instructions of Prof. Otis, we introduce the urethrometer down to the bulbo-membranous junction, and by means of the screw at the handle expand the bulbous extremity "up to a point which is recognized by the patient as filling it (the urethra) completely, and yet easily moving back and forth. The index at the handle then shows the normal circumference of the urethra under examination." This is all beautiful in theory, but very different in practice. If we rely on the patient's feeling his urethra filled by the bulb, we rely on a very unstable guide, because one patient may consider his urethra filled as soon as it is touched by the expanding bulb, while another will not consider his urethra filled until the pain of distension forces an admission from him; and a patient may at different parts of his urethra, according to its tenderness, form different estimates as to when it is distended. The only reliable indication of the distension of the urethra is by moving the urethrometer along the urethra and feeling whether it is held or not, and this may necessitate considerable moving back and forth of the instrument, exposing the urethra to considerable irritation. A contraction is recognized by feeling an obstruction to the withdrawal of the urethrometer when the bulbous portion is reduced just far enough to slip through the contraction; at the same time a note is taken of the size and depth of the stricture. Having passed through the stricture, the bulb is again enlarged until the urethra is filled and so on.

All this requires considerable skill, and a delicacy of manipulation which is seldom attained by any but the expert, accustomed to the handling of urethral instruments. In the hands of an expert the urethrometer may give satisfaction, but in other hands it will perhaps be oftener a source of embarrassment than a source of information.

To my mind the requirements of an ideal instrument for the detection of large strictures would be an instrument the use of which did not require any special skill, and which would make an accurate record of the urethra at all parts. In considering how an instrument could be made to fulfill these requirements, it became evident that the *tactus eruditus* of the surgeon must be replaced by a mechanical contrivance, and that instead of an index on the instrument, a diagram representing the size of the urethra at all parts must be substituted. Acting on these principles, I have had an instrument constructed which I have called a urethrograph. It consists of a canula, terminating in two movable blades, which present a smooth convex surface to the urethra. This instrument is introduced with the blades closed as far as the bulbo-membranous junction; then with the left hand the carriage containing a strip of cardboard is introduced into the slot in the urethrograph. With the index finger of the right hand a movable pin is touched, which liberates a spring contained within the handle of the instrument. This spring, on being liberated, expands the movable blades until a certain adjustable pressure is exerted against the urethral walls. The carriage is now held stationary with the left hand, and with the right the instrument is withdrawn from the urethra. The spring within the handle is so adjusted that the movable blades press against the urethra with an equal pressure at all portions, whether it is strictured or not. As the movable blades move along the urethral wall, they follow its contour, no matter how irregular its shape, and at the same time an arrangement underneath the handle draws a diagram on the cardboard corresponding exactly with the width of the urethra at all parts. This cardboard is spaced longitudinally into millimetres, and transversely into inches, so that at a glance the size and position of any portion of the urethra can be seen. The advantages claimed for this instrument are:

1. The rapidity which an examination of the urethra can be made.

2. The simplicity of the examination, so that it offers no greater difficulties than the introduction of a sound.

3. The accuracy of the results.

4. A uniform pressure is exerted against the urethral wall at all parts, so that the patient does not suffer from the instrument being obstructed in passing through a stricture.

5. This pressure can be adjusted at the will of the operator.

6. A record is obtained so that at a glance the condition of the urethra at any part may be ascertained.

7. These records may be filed away and kept for future reference.

